# Metagenomic insight to apprehend the fungal communities associated with leaf blight of Welsh onion in Taiwan

**DOI:** 10.3389/fpls.2024.1352997

**Published:** 2024-02-29

**Authors:** Himanshi Jayasinghe, Hao-Xun Chang, Stephen Knobloch, Shan-Hua Yang, D. P. Bhagya Hendalage, Kahandawa G. S. U. Ariyawansa, Po-Yu Liu, Marc Stadler, Hiran A. Ariyawansa

**Affiliations:** ^1^ Department of Plant Pathology and Microbiology, National Taiwan University, Taipei, Taiwan; ^2^ Department of Food Technology, Fulda University of Applied Sciences, Fulda, Germany; ^3^ Institute of Fisheries Science, National Taiwan University, Taipei, Taiwan; ^4^ Department of Plant Sciences, Faculty of Science, University of Colombo, Colombo, Sri Lanka; ^5^ School of Medicine, College of Medicine, National Sun Yat-Sen University, Kaohsiung, Taiwan; ^6^ Department Microbial Drugs, Helmholtz Centre for Infection Research GmbH (HZI), Braunschweig, Germany

**Keywords:** alpha-diversity, amplicon sequencing, beta-diversity, co-occurrence networks, pathobiome, phyllosphere, rhizosphere

## Abstract

Plants are associated with a large diversity of microbes, and these complex plant-associated microbial communities are critical for plant health. Welsh onion (*Allium fistulosum* L.) is one of the key and oldest vegetable crops cultivated in Taiwan. The leaf of the Welsh onion is one of the famous spices in Taiwanese cuisine, thus, it is crucial to control foliar diseases. In recent years, Welsh onion cultivation in Taiwan has been severely threatened by the occurrence of leaf blight disease, greatly affecting their yield and quality. However, the overall picture of microbiota associated with the Welsh onion plant is still not clear as most of the recent etiological investigations were heavily based on the isolation of microorganisms from diseased plants. Therefore, studying the diversity of fungal communities associated with the leaf blight symptoms of Welsh onion may provide information regarding key taxa possibly involved in the disease. Therefore, this investigation was mainly designed to understand the major fungal communities associated with leaf blight to identify key taxa potentially involved in the disease and further evaluate any shifts in both phyllosphere and rhizosphere mycobiome assembly due to foliar pathogen infection by amplicon sequencing targeting the Internal Transcribed Spacer (ITS) 1 region of the rRNA. The alpha and beta-diversity analyses were used to compare the fungal communities and significant fungal groups were recognized based on linear discriminant analyses. Based on the results of relative abundance data and co-occurrence networks in symptomatic plants we revealed that the leaf blight of Welsh onion in Sanxing, is a disease complex mainly involving *Stemphylium* and *Colletotrichum* taxa. In addition, genera such as *Aspergillus, Athelia* and *Colletotrichum* were abundantly found associated with the symptomatic rhizosphere. Alpha-diversity in some fields indicated a significant increase in species richness in the symptomatic phyllosphere compared to the asymptomatic phyllosphere. These results will broaden our knowledge of pathogens of Welsh onion associated with leaf blight symptoms and will assist in developing effective disease management strategies to control the progress of the disease.

## 1 Introduction

All plants on Earth harbor a diverse set of microorganisms, including many species from the kingdom of fungi, which play different roles in plants, ranging from beneficial, and neutral to detrimental. The mycobiome represents all the fungal communities that inhabit the particular microhabitat of the plant, in the plant metagenome (Chen et al., 2022). Pathogen invasion is one of the most important biotic factors affecting plant microbiome assembly (Kaushal et al., 2020; López et al., 2020; Yang et al., 2020). Recent studies have revealed that in nature, certain diseases involve the interaction or cooperation of various pathogens causing complex diseases (Lamichhane and Venturi, 2015). Consequently, the concept of ‘pathobiome’ has emerged, challenging the traditional ‘one pathogen–one disease’ hypothesis, which proves insufficient to explain processes involved in complex diseases (Mannaa and Seo, 2021). Studies on plant pathobiomes contribute significantly to identifying not only the pathogens responsible for symptoms but also other microbial species that collaborate with the primary pathogen (Agler et al., 2016). For example, Musonerimana et al. (2020) evaluated the role of the pathogens causing symptoms of rice sheath rot in Burundi and found that rice sheath rot is a complex disease caused by diverse pathogens under different environmental conditions. In their study, Musonerimana et al. (2020) found that *Pseudomonas fuscovaginae* was associated with the symptoms in the highland areas, whereas *Sarocladium oryzae* was associated with those in the lowland areas, especially in the wet season, indicating that these pathogens can independently cause similar sheath rot symptoms. Moreover, few symptomatic samples from lowlands were found to be associated with a high abundance of several well-known phytopathogens such as *Bipolaris* and *Fusarium*, while the abundance of *Sarocladium* in those samples was very low. Thus, exploring the members of the pathobiome is important for understanding the pathogenesis, persistence, transmission, and evolution of plant pathogens to develop microbiome-based disease control strategies (Schlaeppi and Bulgarelli, 2015; Bez et al., 2021). Apart from that, next-generation sequencing (NGS) technology has also broadened our understanding of early detection of plant diseases (Yurgel et al., 2023). For instance, a recent study has found significant differences in oomycete communities between asymptomatic and symptomatic Kiwifruit plants affected by Kiwifruit vine decline syndrome in Italy. The results of Yurgel et al. (2023) showed that *Phytophthora sojae* is the dominant taxa found in symptomatic plants while some other newly identified oomycete species such as *Dactylonectria macrodidyma, Phytopythium citrinum*, and *Thielaviopsis basicola* are also associated with the disease (Savian et al., 2022).

Welsh onion is a popular and economically important crop globally, which is widely used as a spice, vegetable, and even as a medicinal plant worldwide. In Taiwan, Sanxing township in Yilan County is one of the major Welsh onion-growing areas in Taiwan (Wang et al., 2021, Wang et al., 2023). However, recently Welsh onion cultivation in Sanxing has been seriously affected by leaf blight symptoms causing significant yield losses. Recently, Wang et al. (2021) found *Stemphylium vesicarium* species from Welsh onion fields in Sanxing, which can cause leaf blight symptoms in Welsh onion. Nevertheless, it has been reported that other fungal taxa such as *Colletotrichum* spp. causing anthracnose (Yuan et al., 2023; Yu et al., 2023), *Alternaria porri* causing purple blotch (Chang and Huang, 1998) and *Puccinia allii* causing rust are also associated with leaf blight symptoms of Welsh onion in Taiwan (Tzean et al., 2019; Wang et al., 2021). These observations indicate that etiology of leaf blight disease of Welsh onion in Taiwan would be better interpreted by the concept of pathobiome rather than by a simplified model focusing on a single pathogen, given the absence of a single dominant taxon (Mannaa and Seo, 2021).

Even though the recent discoveries regarding fungal pathogens linked with leaf blight of Welsh onion, the knowledge about the interaction between the diversity of the phyllospheric and rhizospheric microbiota in asymptomatic and symptomatic Welsh onion plants naturally observed under field conditions has been poorly studied. In Taiwan, bacterial and fungal communities associated with healthy Welsh onion plants have recently been studied based on culture-dependent method by Wang et al. (2023). Wang et al. (2023) identified *Bacillus*, *Burkholderia*, and *Klebsiella* as the most dominant bacterial genera while *Chaetomium*, *Colletotrichum*, and *Aspergillus* as the predominant fungal genera. Nevertheless, culture-dependent methods display only a very narrow percentage of the entire microbial variability within a sample (Abdelfattah et al., 2018). On the other hand, with recent advances in NGS technology, it has become possible to unravel the diversity of the plant microbiome and its role in plant health and stress tolerance (Ares et al., 2021). In fact, metabarcoding can be a valuable method for evaluation of the diversity of microbes present in phyllosphere and rhizosphere and for recognition of key causal agents responsible in the context of complex diseases such as leaf blight as it delivers a broad image of the genetic variability existent in a sample (Abdelfattah et al., 2018). This method has been widely used in recent investigations to understand microbial communities associated with various plant diseases (Bekris et al., 2021; Dastogeer et al., 2022; Qiu et al., 2022).

In the present study, we hypothesized that fungal community composition would differ between asymptomatic and symptomatic Welsh onion, and that key fungal taxa or groups could serve as early indicators of leaf blight symptoms. Thus, we used high-throughput amplicon sequencing to investigate and compare the structure and composition of fungal communities in the phyllosphere and rhizosphere of Welsh onion affected by leaf blight symptoms to identify the key taxa that might be related to leaf blight diseases of Welsh onion under natural field conditions.

## 2 Materials and methods

### 2.1 Sample collection and preprocessing

Asymptomatic and symptomatic Welsh onion plants of ‘Si-Ji-Cong’ cultivar were collected during early March 2022 from three commercial Welsh onion fields in Sanxing township, Yilan county, where the incidence of leaf blight symptoms had been more than 90% since 2018 (Field 1, 24°40’50.3”N 121°40’23.1”E; Field 2, 24°40’44.4”N 121°40’19.2”E and Field 3, 24°40’12.9”N 121°38’04.1”E) (Wang et al., 2021; Yu et al., 2023) (**Supplementary Figure S1**). Asymptomatic plants with green leaves largely free of chlorotic and necrotic symptoms were collected, whereas symptomatic plants with leaves exhibiting extensive chlorotic and necrotic areas were collected (**Supplementary Figure S2**). Plants at the same growth stage (fourth-true-leaf stage), were collected in order to avoid growth dependent variations in microbial community structures. In total, 5 asymptomatic and 5 symptomatic plants were randomly selected from each field. All tools used for sampling in the field were cleaned using 70% ethanol after every time that a sample was collected. All samples were immediately stored on dry ice in sterile polythene bags and brought to the laboratory. A synopsis of the plant part, plant condition, field name, and the sample names used in the study are shown in **Supplementary Table S1**.

The leaves of asymptomatic and symptomatic plants and the rhizosphere soil were targeted for the mycobiome experiments. Leaves and rhizosphere soils were separated prior to DNA extraction. The procedures included the following steps:(1) Rhizosphere samples were composed of soil adhering to the roots (up to 2.5 mm around the root) at a depth of 15-25 cm. Initially, the roots were gently shaken to discard loosely attached bulk soil, and then the adjacent rhizosphere soil was collected by vigorously shaking the root (Koranda et al., 2011). (2) Plant samples were washed under running tap water to remove soil particles for about 5 minutes, thoroughly washed with sterile distilled water and allowed to drain. From all the symptomatic plants, the proximal part of the third leaf (5 cm region) covering the leaf blight symptoms was selected for total DNA extraction. Similarly, the same region was cut from asymptomatic plants in order to reduce the variations in the microbial community due to the plant compartment. Leaf samples were surface disinfected by immersing the samples in 75% ethanol for 30 seconds and rinsing with sterile distilled water for 1 minute to remove the surface epiphytic microbes (Espinoza et al., 2019; Wang et al., 2023). Aliquots of the sterile distilled water (0.1 mL) used for rinsing were spread on potato dextrose agar (PDA) (supplemented with 100 mg/L ampicillin) and incubated at 25°C to check that leaf surfaces were thoroughly disinfected.

### 2.2 Total DNA extraction and amplicon sequencing

Before DNA extraction, approximately 100 mg of plant material and 400 mg of soil sample from each sample were ground into a fine powder in the presence of liquid nitrogen using a sterile mortar and pestle. Total DNA from plant leaves was extracted using the DNeasy plant Minikit (Qiagen, Hilden, Germany) following the user’s manual with slight modifications. Modifications include addition of six autoclaved DNAase-free metal beads (diameter-2.381 mm) (Bioman; Bioman Scientific Co., Ltd., New Taipei, Taiwan) to each tube with leaf powder and AP1 buffer and grinding with the Geno/Grinder homogenizer (USA) at 1500 rpm for 30 minutes at 5-minute intervals and incubating the homogenized samples at 65 °C for one hour while mixing by inverting the tube several times. Similarly, soil gDNA was extracted using the PowerSoil DNA Isolation kit (Qiagen, Hilden, Germany) using a protocol slightly modified from the manufacturer’s recommendations. Modifications to the soil DNA extraction protocol include, increasing the input sample amount up to 400 mg, grinding with the Geno/Grinder homogenizer (USA) at 1500 rpm for 30 minutes at 5-minute intervals, incubating the homogenized samples at 65°C for 1 hour while mixing and washing the pellet with DNA using EA and C5 buffers twice. DNA from leaf and soil samples were eluted in a final volume of 60 µL of ultrapure, sterile double distilled water. Plant and soil DNA quality and quantity were checked both by electrophoresis in 2% (w/v) agarose gels and by using a NanoDrop ND-1000 spectrophotometer (Thermo Fisher Scientific, Waltham, MA, USA).

The ITS1 region (Scibetta et al., 2018) of fungi in Welsh onion leaves was amplified by two-step Polymerase Chain Reaction (PCR) by using newly developed primers (Toju et al., 2012). Two-step PCR with newly developed primers were carried out to reduce the amplification of host plant DNA and maximize the fungal DNA for sequencing. The PCR mixture of the first step contained the following ingredients per sample: 5.0 μL of KAPA HiFi Buffer (5X), 0.75 μL KAPA dNTP Mix (mM), 0.5 μL of KAPA HiFi DNA Polymerase (1 U/μL), 0.75 μL of each primer (10 μM), approximately 2 ng of genomic DNA, and water to a final volume of 25 μL. The second step PCR included 1 μL of the first PCR product as the input, along with all the other ingredients mentioned above in the first step of PCR. The primer pairs and the PCR conditions used for each step are listed in **Supplementary Tables S2** and **S3** respectively. Sequencing of the fungal ITS1 region in the Welsh onion leaves was conducted at Tri-I Biotech, Inc. (New Taipei City, Taiwan) and rhizosphere soil at BIOTOOLS Co., Ltd. (Taipei, Taiwan) companies respectively using Illumina MiSeq 2x 300 bp paired-end sequencing.

### 2.3 Sequence data processing

The quality of the raw reads was assessed with FastQC (Andrews, 2010) and the resulting raw data were analyzed using the QIIME 2 version 2021.11.0 (Bolyen et al., 2019). In QIIME 2, Cutadapt plugin was used to remove primer sequences while DADA2 plugin was applied to filter, trim, denoise, to merge forward and reverse reads and also to remove chimeras (Callahan et al., 2016). Amplicon sequence variants (ASVs) were assembled and taxonomically assigned into different classification levels using VSEARCH consensus taxonomy classifier in QIIME2 (Rognes et al., 2016) based on the UNITE ITS v8.2 reference database (Abarenkov et al., 2020). ASVs not classified at kingdom and phylum level and those classified as mitochondrial and chloroplast sequences were removed from downstream analysis.

All statistical analyses and data visualizations for the resulting ASVs were performed using the R package “phyloseq” v1.42.0 (McMurdie and Holmes, 2013) with ggplot2 package v3.4.2 (Wickham, 2016) in R environment v4.2.2 (R core team, 2022). Rarefaction curves were constructed for the number of observed ASVs using the package vegan v2.6.4 (Oksanen et al., 2020). Venn diagrams were generated to visualize distinct and shared ASVs between asymptomatic and symptomatic plants through the “ps_venn” function in MicEco package v0.9.19 (Russel, 2021). The total count of ASVs and assigned taxa for each taxonomic rank were transformed to relative abundance and visualized using ggplot2 package. Alpha-diversity was measured using the richness, Chao 1, ACE and Shannon indices by the “estimate_richness” function of phyloseq. Non-parametric Kruskal–Wallis and pairwise Wilcoxon rank sum tests were used to identify differences in diversity, species richness, and evenness among the asymptomatic and symptomatic Welsh onion plants across the three fields. A Principal coordinates analysis (PCoA) was performed based on the Bray-Curtis distance. Significant differences in the beta-diversity were tested with non-parametric “adonis2” function of the vegan package with 999 permutations. A linear discriminant analysis (LDA) effect size (LEfSe) with the Kruskal–Wallis and Wilcoxon rank-sum tests, were used to identify the fungal taxa that were significantly different between asymptomatic and symptomatic plants (Segata et al., 2011). Statistical significance was claimed at false discovery rate (FDR) of 0.05 for all Kruskal–Wallis tests and Wilcoxon tests, and the threshold for the LEfSe analysis score was set at 2.0.

Co-occurrence networks of phyllosphere and rhizosphere fungal communities were constructed and compared for asymptomatic and symptomatic samples with the R package “NetCoMi” v1.1.0 using the taxonomic profiles at generic level (Peschel et al., 2021). Covariates from the fields were confounded in the network analysis because of the limitation of the covariate for plant sampling. To simplify the networks, the top 50 most abundant ASVs were used. Correlation network analysis was computed with the SparCC method. Eigenvector centrality was used for scaling node size and picking hub taxa. Cluster patterns were detected by the ‘cluster_fast greedy’ algorithm (Clauset et al., 2004) and nodes with the same colors indicated that they were from the same cluster. Hub taxa were determined using the threshold of 0.95 (Bhandari et al., 2023). Correlation absolute values below 0.3 were filtered out. Networks were compared using 1,000 permutations to estimate statistical differences and the statistical significance was decided at the Benjamini-Hochberg corrected p value of 0.01 (Benjamini and Hochberg, 2000). Similarities and clustering between fungal community networks were measured using the Adjusted Rand Index (ARI) and the Jaccard index (Gates et al., 2019; Kong et al., 2023).

## 3 Results

### 3.1 Fungal community differences in leaves of asymptomatic and symptomatic Welsh onion plants

Mycobiome analysis between asymptomatic and symptomatic Welsh onion plants was performed in order to check how the plant mycobiome can be affected by leaf blight symptoms. Amplicon sequencing of fungal communities in Welsh onion leaves using the Illumina MiSeq platform resulted in a total of 928,226 high-quality reads with an average read length of 301 bp, across both asymptomatic and symptomatic Welsh onion samples in the three sampling locations.

After de-noising and quality filtering by DADA2 in QIIME2, a total of 688,645 sequences were obtained for leaf samples. The sequence details after denoising are given in **Supplementary Table S4**. For the leaf, 254 ASVs that belonged to the kingdom fungi were left after 18 sequences (on average 6.62% of the total ASVs) that are unassigned or belonging to plant chloroplast were removed. The 254 fungal ASVs were classified into three phyla, 13 classes, 26 orders, 60 families and 67 genera. To better understand differences of the fungal communities between these two plant conditions, some general features related to the fungal community structures were analyzed. Initially, the number of shared and unique fungal ASVs between asymptomatic and symptomatic leaves in each field were presented in Venn diagrams (**Supplementary Figure S3**). In field 1, 28 ASVs exclusive to the asymptomatic leaves, and 58 ASVs exclusive to the symptomatic leaves were found. There were only 6 ASVs shared between the asymptomatic and symptomatic leaves. In field 2, 47 ASVs were found only in asymptomatic leaves, whereas 11 ASVs were found exclusively in symptomatic leaves. There were 25 ASVs were shared between both symptomatic and asymptomatic leaves in field 2. In field 3, 35 ASVs exclusive to the asymptomatic leaves, and 87 ASVs exclusive to the symptomatic leaves. There were only 9 ASVs shared between both asymptomatic and symptomatic leaves in field 3.

**Figure 1:**
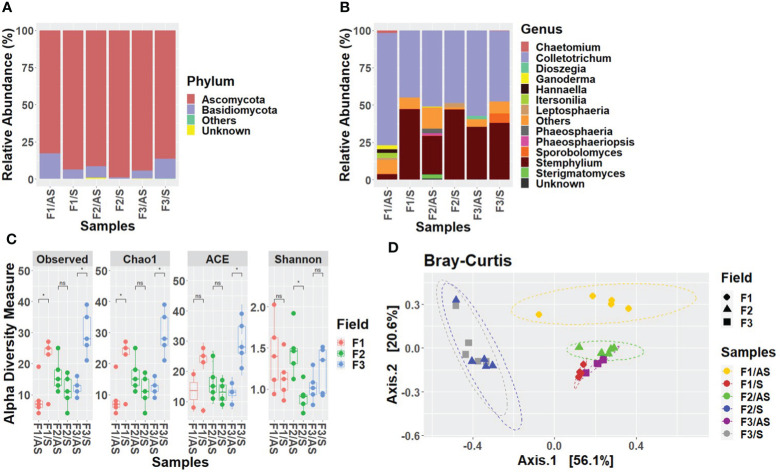
Relative abundance of the fungal communities in Welsh onion leaves. Stacked bar charts show the relative abundance divided according to the plant condition and the field. **(A)** at phylum level, **(B)** at genus level. Taxa whose abundance was < 0.25% have been grouped into ‘Others’ category; taxa that did not classify into a specific taxonomic level were grouped as ‘Unknown’. **(C)** Alpha-diversity estimations of the fungal communities in Welsh onion leaves based on Observed, Chao1, ACE and Shannon indices. Box plot depicts median (central horizontal lines) and the inter-quartile ranges (boxes). Asterisks indicate significant differences between two groups of samples based on Wilcoxon-test. * denotes *p* < 0.05, whereas ‘ns’ denotes no significant difference as determined by Wilcoxon-test (P-value <0.05). **(D)** Beta-diversity analysis of the fungal communities in Welsh onion leaves. PCoA plot is based on the Bray–Curtis distances between symptomatic and asymptomatic plants in three different fields. Ellipses show confidence intervals **(CI)** of 95% for each sample type. Statistical significance has been inferred using PERMANOVA.

To determine the differences in fungal community abundance between the asymptomatic and symptomatic samples, the relative abundance of fungi in each sample was calculated. At the phylum level, the mycobiome of Welsh onion leaves in all three fields was overall dominated mainly by Ascomycota and Basidiomycota. In fields 1 and 2, the relative abundance of Ascomycota was lower in asymptomatic samples (field 1: 82%, field 2: 92%) compared to symptomatic samples (field 1: 93%, field 2: 99%), whereas the relative abundance of Basidiomycota was higher in asymptomatic samples (field 1: 18%; field 2: 8%) compared to symptomatic samples (field 1: 7%, field 2: 1%). Nevertheless, an opposite pattern was observed in field 3, where the relative abundance of Ascomycota was high in asymptomatic samples (94%) compared to symptomatic samples (87%), and the relative abundance of Basidiomycota was low in asymptomatic samples (6%) compared to symptomatic samples (12%) (**Figure 1A**). At the class level, the most prevalent fungal classes in both symptomatic and asymptomatic Welsh onion leaves were Agaricomycetes, Dothideomycetes, Microbotryomycetes, Sordariomycetes, and Tremellomycetes. In all three fields, percentages of Sordariomycetes and Agaricomycetes was high in asymptomatic plants (field 1: 73%, field 2: 54%, field 3: 59% and field 1: 9%, field 2: 4%, field 3: 3%) whereas their percentage was comparatively low in symptomatic plants (field 1: 45%, field 2: 48%, field 3: 48% and field 1: 0%, field 2: 0%, field 3: 0%). On the contrary, the percentage of Dothideomycetes was high in symptomatic plants (48%, 51% and 39%) compared to asymptomatic plants (9%, 36% and 35%) in field 1, 2 and 3 respectively. Furthermore, members of the classes Agaricostilbomycetes and Malasseziomycetes were unique to the asymptomatic plants of fields 2 and 1 respectively (**Supplementary Figure S4**). At the order level, the most abundant fungal orders were Glomerellales, Pleosporales and Polyporales. In all three fields, the percentage of Glomerellales and Polyporales was high in asymptomatic plants (field 1: 71%, field 2: 49%, field 3: 57% and field 1: 9%, field 2: 2%, field 3: 3%), whereas their percentage was comparatively low in symptomatic plants (field 1: 45%, field 2: 48%, field 3: 48% and field 1: 0%, field 2: 0%, field 3: 0%). On the contrary, the percentage of Pleosporales was high in symptomatic plants (48%, 51%, 39%) compared to asymptomatic plants (9%, 36%, 35%) in fields 1, 2 and 3 respectively. Furthermore, fungal orders such as Cystofilobasidiales, Malasseziales and Sordariales were found only in asymptomatic plants of field 2 (with the relative abundance of 3%, 3% and 2% respectively) (**Supplementary Figure S5**). At the family level, the most predominant taxa in both asymptomatic and symptomatic Welsh onion leaves were clustered in Pleosporaceae, Glomerellaceae and Phaeosphaeriaceae. In all three fields, the relative abundance of Glomerellaceae, and Phaeosphaeriaceae was high in asymptomatic plants (field 1: 71%, field 2: 49%, field 3: 57% and field 1: 4%, field 2: 11%, field 3: 1%) where as their percentage was comparatively low in symptomatic plants (field 1: 45%, field 2: 48%, field 3: 48% and field 1: 0%, field 2: 0%, field 3: 0%) (**Supplementary Figure S6**). At the genus level, *Stemphylium* and *Colletotrichum* were the most predominant genera in all the samples, collectively constituting more than 90% of the identified amplicons. The abundance of *Stemphylium* was high in symptomatic samples (47%, 48%, 38%) compared to asymptomatic samples (3%, 25%, 35%) whereas the percentage of *Colletotrichum* was high in asymptomatic plants (71%, 49% and 57%) compared to symptomatic plants (45%, 48%, 48%) in field 1, 2 and 3, respectively. Considering the fungi in asymptomatic plants, *Chaetomium, Hannaella* and *Itersonilia* were found only in field 1 (2%, 3% and 3%) while *Phaeosphaeria* and *Sterigmatomyces* only in field 2 (3%, 2%) and *Dioszegia* in field 3 (2%). The genus *Ganoderma* was found in asymptomatic plants in both fields 1 (3%) and 2 (1%). Considering the fungi in symptomatic plants, *Sporobolomyces* was only found in the asymptomatic plants of field 3 (6%) (**Figure 1B**).

**Figure 2:**
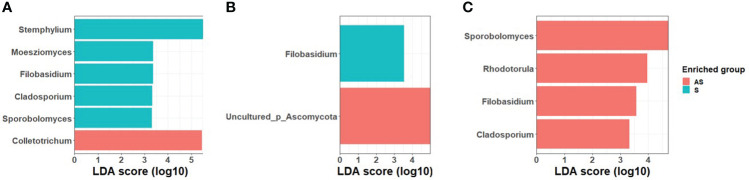
LDA scores of fungal taxa enrichment between asymptomatic and symptomatic leaves of Welsh onion in **(A)** Field 1, **(B)** Field 2, **(C)** Field 3. Only genera with a *P* value < 0.05 for the Kruskal–Wallis test and an LDA score > 2 are displayed.

LEfSe analysis was used to search for statistically significant taxonomic and functional biomarkers between asymptomatic and symptomatic leaf samples (**Figure 2**). In field 1, the fungal genera *Cladosporium, Filobasidium, Moesziomyces, Sporobolomyces* and *Stemphylium* were statistically enriched in symptomatic leaves while *Colletotrichum* was statistically enriched in asymptomatic leaves. In field 2, *Filobasidium* was significantly abundant in symptomatic samples while an unidentified taxon belonging to Ascomycota (Uncultured_p_Ascomycota) was significantly abundant in symptomatic leaves. In field 3, contrary to field 1 and 2, *Cladosporium, Filobasidium, Rhodotorula*, and *Sporobolomyces* were significantly enriched in asymptomatic plants whereas none of the fungal genera were significantly enriched in symptomatic samples.

Rarefaction curves based on the number of fungal species reached a plateau when increasing the number of samples, indicating that the sampling depth was sufficient to cover the actual fungal diversity within the samples (**Supplementary Figure S7**).

Alpha and beta-diversity metrics were calculated to assess the effects of the plant condition and field location on Welsh onion mycobiota assembly. In terms of alpha-diversity indices, symptomatic plants had higher species richness than asymptomatic plants in fields 1 and 3. In fields 1 and 3, except for the ACE index in field 3. As for Shannon, field 3 was statistically significant higher in asymptomatic leaves compared to symptomatic leaves. (**Figure 1C**). To evaluate the differences in the fungal composition of all symptomatic and asymptomatic plants in the three fields, PCoA was performed based on Bray-Curtis distances. The PERMANOVA showed that there is a significant difference in putative ASVs between symptomatic and asymptomatic samples in each field location (*R* ^2^ = 0.70, *P* < 0.0001) (**Figure 1D**).

**Figure 3:**
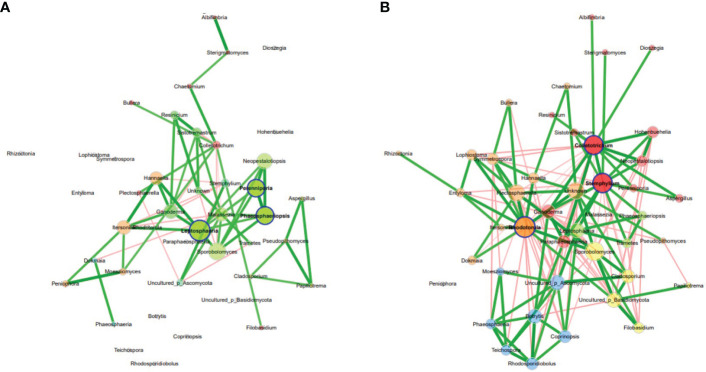
Co-occurrence network analysis of fungal communities in Welsh onion leaves. **(A)** asymptomatic samples, **(B)** symptomatic samples. Hubs are highlighted by bold borders. Node sizes were determined with eigenvector centrality. Node color indicates the cluster determined by greedy modularity optimization. Edge weights depict similarities between nodes. Edge color represents positive (green) and negative (red) correlations.

Analysis of co-occurrence networks of fungal communities in the Welsh onion leaves indicated that most of the global network properties were almost similar between the asymptomatic and symptomatic leaves except for the edge density, modularity, and average dissimilarity which were significantly different between the symptomatic and asymptomatic leaves (**Supplementary Table S5**). The modularity was 0.75 and 0.40 for asymptomatic and symptomatic leaves, respectively, indicating a relatively high modularity (Newman, 2006). Networks had an average path length of 1.59 and 1.65 and contained 89% and 73% positive edges for asymptomatic and symptomatic leaves, respectively. However, the betweenness centrality, eigenvector centrality and hub taxa of the most influential nodes and the cluster patterns (ARI = -0.003, P value < 1.000) were not significantly different between the two samples (**Figure 3**) (**Supplementary Table S6**). The unique hubs in the asymptomatic network included fungal genera (*Leptosphaeria, Perenniporia and Phaeosphaeriopsis*) while that of the symptomatic network included fungal genera (*Colletotrichum, Paraphaeosphaeria and Stemphylium*). Additionally, the most abundant taxa (*Colletotrichum, Stemphylium and Rhodotorula*) in the Welsh onion leaves were hubs in symptomatic networks, suggesting that hub taxa may be important for symptom development. Moreover, the number of connections (i.e., edges) in fungal communities of the symptomatic leaves was higher than in the asymptomatic leaves (**Supplementary Table S5**).

### 3.2 Fungal community differences in rhizosphere of asymptomatic and symptomatic Welsh onion plants

A total of 2,927,657 high quality raw sequences with an average read length of 247 bp were generated from rhizosphere samples of asymptomatic and symptomatic plants in the three sampling locations. After de-noising and quality filtering by DADA2 in QIIME2, a total of 2,418,353 sequences were obtained. The sequence details after denoising are given in **Supplementary Table S7**. Venn diagrams showed that, in field 1 and 2, 1005 and 1075 ASVs exclusive to the asymptomatic samples, 387 and 353 ASVs detected only in symptomatic samples, and 357 and 331 ASVs shared between asymptomatic and symptomatic samples respectively. In field 3, asymptomatic samples and symptomatic samples had 629 and 751 unique ASVs respectively while 243 ASVs were shared between symptomatic and asymptomatic samples (**Supplementary Figure S8**). For soil, 4054 fungal ASVs were left for analysis after 100 non-fungal sequences (on average 2.40% of the total ASVs) were removed. The taxonomic assignment of the fungal ASVs in soil revealed 9 phyla, 30 classes, 88 orders, 312 families and 307 genera in all the samples.

**Figure 4:**
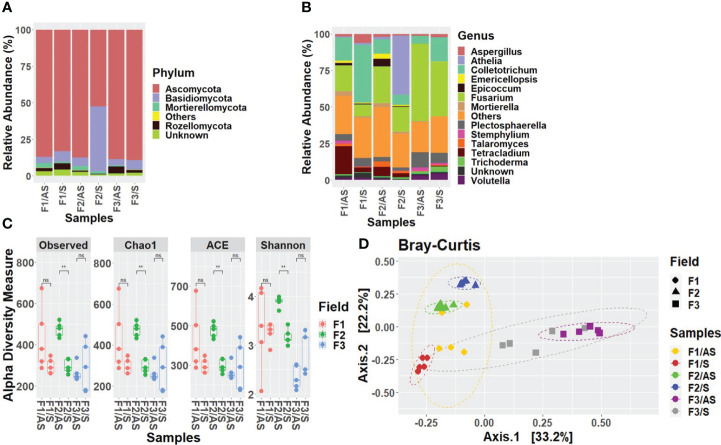
Relative abundance of the fungal communities in Welsh onion rhizosphere. Stacked bar charts show the relative abundance divided according to the symptomatic state and the field. **(A)** at phylum level. **(B)** at genus level. Taxa whose abundance was < 1% have been grouped into ‘Others’ category; taxa that did not classify into specific taxonomic level was grouped as ‘Unknown’. **(C)** Alpha-diversity estimations of the fungal communities in Welsh onion rhizosphere based on observed, Chao1, ACE and Shannon indices. Box plot depict median (central horizontal lines) and the inter-quartile ranges (boxes). Asterisks indicate significant differences between two groups of samples based on Wilcoxon-test. ** denotes *p* < 0.01, whereas ‘ns’ denotes no significant difference as determined by Wilcoxon-test (P-value <0.05). **(D)** Beta-diversity analysis of the fungal communities in Welsh onion rhizosphere. PCoA plot is based on the Bray–Curtis distances between asymptomatic and symptomatic plants in three different fields. Ellipses show confidence intervals (CI) of 95% for each sample type. Statistical significance has been inferred using PERMANOVA.

Results indicated that the fungal species in the rhizosphere samples were more diverse and differed in their relative abundance with regard to the plant condition and field location. At the phylum level, rhizosphere samples of asymptomatic and symptomatic plants across the 3 fields were dominated by Ascomycota followed by Basidiomycota, Mortierellomycota and Rozellomycota. In fields 1 and 2, the abundance of Ascomycota was higher in asymptomatic plants (field 1: 87%, field 2: 88%) compared to symptomatic plants (field 1: 83%, field 2: 53%), whereas in field 3, its percentage was low in symptomatic plants (89%) compared to asymptomatic plants (88%). Interestingly, the relative abundance of Basidiomycota was relatively high in symptomatic plants in field 2 compared to any other samples (44%) (**Figure 4A**). In the Welsh onion rhizosphere, fungal classes such as Agaricomycetes, Dothideomycetes, Eurotiomycetes, Leotiomycetes and Tremellomycetes were more abundant. The percentage of Agaricomycetes was high in symptomatic samples compared to asymptomatic samples in all three fields, while in symptomatic plants in field 2, it was remarkably high (42%) compared to other fields. In fields 1 and 2, the percentage of Dothideomycetes was higher in asymptomatic samples (field 1: 11%, field 2: %) compared to symptomatic samples (field 1: 8%, field 2: 4%), while in field 2, its percentage was lower in asymptomatic samples (9%) compared to symptomatic samples (11%) (**Supplementary Figure S9**). The most predominant fungal orders in all the samples were Atheliales, Eurotiales, Glomerellales, Helotiales, Hypocreales, Pleosporales, and Sordariales. Among those, the percentage of Atheliales was high in symptomatic plants in field 2 (41%) compared to the other two fields. Apart from that, the percentage of Glomerellales was remarkably high in field 1 compared to the other two fields, and when comparing its abundance between the two symptomatic states, its percentage was higher in symptomatic plants (46%) compared to asymptomatic plants (21%) (**Supplementary Figure S10**). At the family level, several fungal families such as Aspergillaceae, Atheliaceae, Glomerellaceae and Nectriaceae were more abundant. Among those the relative abundance of Atheliaceae was very high in symptomatic plants of field 2, compared to other fields (41%). The percentage of Nectriaceae was high in asymptomatic plants (19%, 26%, 56%) compared to symptomatic plants (9%, 17%, 41%) in field 1, 2 and 3 respectively (**Supplementary Figure S11**). At the generic level, the most abundant fungal genera were *Athelia, Aspergillus, Colletotrichum, Epicoccum, Fusarium, Plectosphaerella, Talaromyces* and *Trichoderma.* The genus *Colletotrichum* was detected in high abundance in field 1 compared to other fields, and its percentage was high in asymptomatic plants (40%) compared to symptomatic (16%) plants, while *Athelia* was highly abundant in the symptomatic plants of field 2 compared to other samples (41%). In addition to that, in all three fields, the genus *Fusarium* was more abundant in asymptomatic plants (field 1: 17%, field 2: 25%, field 3: 52%) compared to symptomatic plants (field 1: 8%, field 2: 17%, field 3: 37%) (**Figure 4B**).

**Figure 5:**
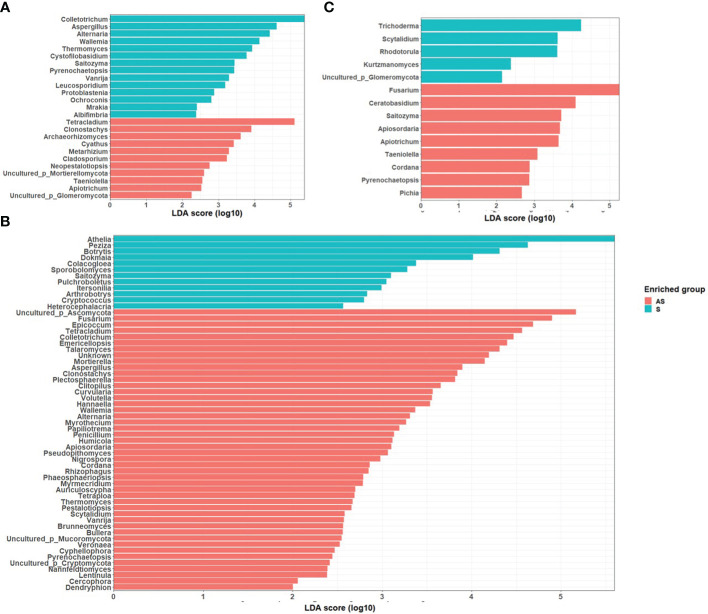
LDA scores of fungal taxa enrichment between rhizosphere of asymptomatic and symptomatic plants in **(A)** Field 1, **(B)** Field 2, **(C)** Field 3. Only genera with a *P* value < 0.05 for the Kruskal–Wallis test and an LDA score > 2 are displayed.

LEfSe identified several fungal taxa that were specifically enriched in different fields under the asymptomatic and symptomatic conditions. Notably, in field 1, the fungal genera *Alternaria, Aspergillus* and *Colletotrichum* were significantly enriched in rhizosphere soils of symptomatic plants, while *Clonostachys, Cyathus, Metarhizium*, and *Tetracladium* were statistically enriched in asymptomatic plants. In field 2, *Athelia, Botrytis*, and *Peziza* were statistically abundant in symptomatic plants while *Epicoccum, Fusarium, Mortierella* and *Talaromyces* were statistically abundant in asymptomatic plants. In field 3, the statistically significant genera in symptomatic samples were *Rhodotorula*, *Scytalidium* and *Trichoderma* while in asymptomatic plants were *Ceratobasidium, Fusarium* and *Saitozyma* (**Figure 5**).

Rarefaction curves showed that all rhizosphere soil samples of asymptomatic and symptomatic plants were near saturation, indicating that most ASVs from the rhizosphere fungal communities were detected (**Supplementary Figure S12**).

In the case of the Welsh onion rhizosphere, species richness (Observed, Chao1 and ACE) was high in asymptomatic plants compared to symptomatic plants in fields 1 and 2 and it is statistically significant in field 2. In regard to Shannon diversity, higher Shannon diversity was observed in asymptomatic plants in fields 1 and 2 and the difference in symptomatic state was statistically significant in field 2 (**Figure 4C**). In the Welsh onion rhizosphere samples, PCoA revealed a significant difference of fungal communities between asymptomatic and symptomatic samples in each field location (PERMANOVA, *R* ^2^ = 0.71, *P* < 0.001) (**Figure 4D**).

**Figure 6:**
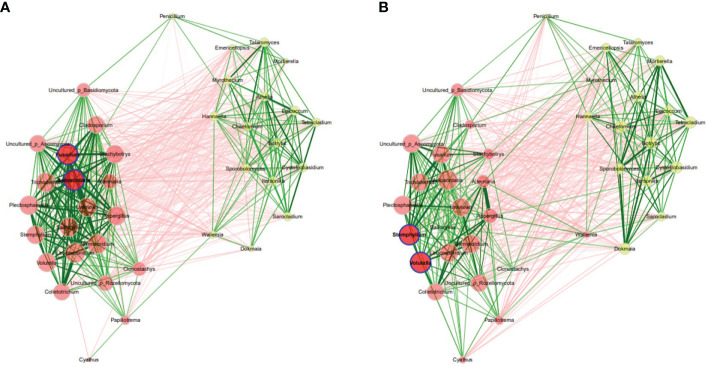
Co-occurrence network analysis of fungal communities in Welsh onion rhizosphere. **(A)** asymptomatic samples, **(B)** symptomatic samples. Hubs are highlighted by bold borders. Node sizes were determined with eigenvector centrality. Node color indicates the cluster determined by greedy modularity optimization. Edge weights depict similarities between nodes. Edge color represents positive (green) and negative (red) correlations.

In rhizosphere, global network properties resulted from network analysis indicated that the properties were similar between the fungal communities in asymptomatic and symptomatic leaves (**Supplementary Table S8**). The modularity was 0.006 and 0.055 for rhizosphere of asymptomatic and symptomatic plants respectively, indicating a relatively high modularity. Networks had an average path length of 1.08 and 1.15 and contained 50% and 49% positive edges for rhizosphere of asymptomatic and symptomatic plants, respectively. However, the betweenness centrality, eigenvector centrality and hub taxa of the most influential nodes and the cluster patterns (ARI = 0.365, P value < 0.05) were significantly different between asymptomatic and symptomatic conditions (**Figure 6**) (**Supplementary Table S9**). The unique hubs in the fungal network of symptomatic plants included fungal genera (*Fusarium and Apiosordaria*), while that of the asymptomatic plants included fungal genera (*Stemphylium and Volutella)*. Additionally, some of the abundant taxa in the rhizosphere of Welsh onion plants (*Athelia and Colletotrichum*) were not hubs in networks, suggesting that other taxa besides those most abundant may be associated with the rhizosphere of Welsh onion plants. However, the number of connections (i.e., edges) in the symptomatic plants were less than in the asymptomatic plants (**Supplementary Table S8**).

## 4 Discussion

In this study, a comprehensive view of the naturally occurring Welsh onion-associated mycobiome in three different Welsh onion fields and their variations in response to leaf blight symptoms was evaluated for the first time. The results indicated that the rhizosphere and phyllosphere mycobiomes of asymptomatic and symptomatic Welsh onion plants have many differences in their overall composition, abundance, and diversity indices. The lower richness and diversity of phyllosphere communities compared to the soil rhizosphere samples was evident from the results, and it is consistent with reports from several other plant species such as *Solanum lycopersicum* L., *Phragmites australis, Stellera chamaejasme* etc (Jin et al., 2014; Dong et al., 2019; Zhou et al., 2019). suggesting that nutrient availability due to the activity of plant root exudates can be the major factor that affects high rhizosphere microbial composition (Qu et al., 2020).

Plant pathobiome studies are valid approaches to investigating the possible pathogenic agents that are causing complex disease symptoms (Musonerimana et al., 2020). In this study, the comparison of the leaf mycobiome of asymptomatic and symptomatic plants in three different field locations indicated the dominance of *Stemphylium* in symptomatic plants, suggesting that the *Stemphylium* is one of the most abundant and core species in the symptomatic plant microbiome. These observations are in accordance with previous studies where *Stemphylium vesicarium* was identified as one of the major foliar pathogens of Welsh onion causing leaf blight symptoms in Taiwan during the period of December to March (Wang et al., 2021). Additionally, it was revealed that potentially pathogenic *Stemphylium* was found not only in symptomatic plants but also in asymptomatic plants. The presence of *Stemphylium* in asymptomatic plants may imply that these plants with no visible symptoms were at the initial stages of disease development, or that their virulence was suppressed due to genetic resistance of the individual plant or their commensal microorganisms (Kovalchuk et al., 2018).

It is worth noting that *Colletotrichum* is also identified as one of the most abundant fungal groups in symptomatic Welsh onion leaves apart from *Stemphylium.* Several species of *Colletotrichum* have been isolated from diseased Welsh onion plants with leaf blight symptoms as well as from asymptomatic leaves as an endophyte in the Sanxing area during our previous studies (Wang et al., 2023; Yu et al., 2023). One plausible explanation for these observations is that certain fungal taxa, such as *Colletotrichum*, can exist as endophytes in the plant and transition to a saprotrophic state with the death of the plant tissue (Promputtha et al., 2007). Another potential scenario is that pathogenic *Colletotrichum* species may undergo a quiescent stage in their lifecycle before symptoms manifest in the host plant (Ranathunge et al., 2012; Mongkolporn and Taylor, 2018). Therefore, in this study, observation of *Colletotrichum* in asymptomatic and symptomatic leaves may be due to the quiescent stage of *Colletotrichum* in Welsh onion or due to a change of its lifestyle from endophytic to saprotrophic nature with the host plant decay. Besides, network identified *Colletotrichum* and *Stemphylium* as hub taxa in the phyllosphere of symptomatic plants. This indicates that, leaf blight disease of Welsh onion in Sanxing might be a complex disease, involving different pathogenic species, causing similar symptoms on a common host plant species. For instance, Le May et al., 2009 indicated that Ascochyta blight in pea plants of France is a complex disease involving two fungal pathogens named *Mycosphaerella pinodes* and *Phoma medicaginis* var. *pinodella.* Le May et al. (2009) further showed that subsequent inoculation of the two pathogens one after the other can increase the disease severity than the simultaneous inoculation of both pathogens. It is evident that presently using fungicides do not offer effective field mitigation of leaf blight diseases of Welsh onion in Sanxing, Taiwan (Wang et al., 2021, Wang et al., 2023; Yu et al., 2023). So based on the results of the present study we speculate that the main reason for this failure relates to *Collectrichum* and *Stemphylium* pathogen complexes involved in causing the leaf blight of Welsh onion. Hence, it is essential to investigate potential fungicides, which can be used to control both of these fungal groups under field conditions to effectively manage the diseases. Moreover, since the season also plays an important role in symptom development in different crops such as rice according to previous studies (Musonerimana et al., 2020), further studies should be conducted as an extension to our study to evaluate the effect of seasonal variations on pathogen viability associated with leaf blight symptoms.

Plant pathobiome studies also help in the identification of other key microbes that interact consistently with the disease-causing pathogens (Musonerimana et al., 2020). The current study found that symptomatic leaves tend to be colonized by a higher number of fungal species, as evidenced by the significantly higher values obtained for fungal richness in symptomatic plants than in asymptomatic plants. A recent study also indicated that the alpha-diversity of fungal communities associated with crabapple species increases with the infection process by the rust fungus *Gymnosporangium yamadae* (Zhang et al., 2023). The increased fungal richness associated with the disease can be due to the fact that the pathogen interferes with the plant immune system, facilitating the entry of numerous microbes into the plant to compete for the available resources (Hu et al., 2020). Many of the microbial genera in symptomatic leaves, such as *Gymnopilus* (Vohník and Réblová, 2023), *Rhizoctonia* (Masuhara et al., 1993), *Phaeosphaeria* (Minter and Cannon, 2017) have been reported to contain saprobic species according to previous studies, indicating that opportunistic fungal species might have colonized the plants after the pathogen infection. Similarly, a previous study demonstrates that some of the Norway spruce trees naturally infected by *Heterobasidion* spp. have been co-infected with other saprobic and wood-degrading microbes (*Amylostereum areolatum, Inonotus* sp., *Penicillium* sp., *Stereum sanguinolentum*, *Talaromyces* sp., *Trichoderma atroviridis*) after the infection of *Heterobasidion* spp (Kovalchuk et al., 2018). *Athelia* is a broad host range pathogen that causes various diseases in plants, like stem rot in peanuts (Yan et al., 2022), southern blight in common bean (Paul et al., 2023) and collar rot in soybean (Zheng et al., 2021). It has been found that *A. rolfsii* can cause white rot in onions (Konjengbam and Devi, 2022). *Aspergillus* is also found in relatively high amounts in symptomatic plants compared to asymptomatic plants in fields 1 and 3. Several species of *Aspergillus* are common agricultural pests. For instance, a recent metagenomic study indicates that the rhizosphere of maize plants infected by northern corn leaf blight is composed of more *Aspergillus* species compared to an asymptomatic rhizosphere (Dlamini et al., 2023). Even though *Colletotrichum* is a foliar pathogen, in the present study *Colletotrichum* species were found in the rhizosphere soil of symptomatic plants, which may be because of the availability of *Colletotrichum* spores in soil due to the long-term cultivation of Welsh onion under highly symptomatic conditions during both summer and winter. These evidences support the pathobiome concept indicating that Welsh onion with leaf blight symptoms can be co-infected with different opportunistic pathogens.

*Fusarium* species are known to cause various disease symptoms in Welsh onion. In Japan, *Fusarium oxysporum* has been identified as the causal agent of basal rot in Welsh onion (Alberti et al., 2018). Meanwhile *Fusarium proliferatum* has been identified from Welsh onion undergoing basal stem rot in Italy (Dissanayake et al., 2009). However, it has also been reported that certain *Allium* species possess resistance to pathogenic *Fusarium* species. For instance, a study conducted in the Netherlands has found that *Allium fistulosum* and *A. schoenoprasum* show high levels of resistance to Fusarium basal rot caused by *F. oxysporum* and *F. proliferatum* isolates (Galván et al., 2008). Thus, in this study higher abundance of *Fusarium* associated with asymptomatic plants without showing any symptoms of rotting or wilting, gives some evidence that Welsh onion plants in Sanxing are resistant to any symptom caused by *Fusarium* species. In addition to that, results of this study indicated that the genus *Mortierella* was significantly abundant in the rhizosphere of asymptomatic Welsh onion plants. In fact, similar results were observed in another study where *Mortierella* was dominant in healthy Bayberry trees, not affected by decline disease (Ren et al., 2021). Wang et al., 2022 demonstrated that inoculation of *Mortierella alpina* to *Panax ginseng* plants infected with *Fusarium oxysporum* significantly controlled the pathogen while stimulating the plant to recruit more plant growth-promoting bacteria (*Pseudomonas*, *Rhizobium* and *Sphingomonas*). The occurrence of these beneficial fungal species specifically associated with asymptomatic plants may give hints about their role in pathogen suppression.

The alpha-diversity analysis showed that the rhizosphere soil samples from asymptomatic Welsh onion plants had higher fungal diversity than the symptomatic samples. Similar observations were reported by Wu et al., 2015 where they identified lower alpha-diversity associated with a diseased rhizosphere compared with a healthy rhizosphere in *Panax notoginseng* plants affected by root rot disease in China. A higher diversity of rhizosphere fungi in healthy plants often leads to greater resistance against pathogens due to the complex interactions among the microorganisms in the soil (Wei et al., 2021). Additionally, lower diversity in the symptomatic rhizosphere may be due to the limited availability of carbon for microbes in the rhizosphere affecting the growth of the microbes (Ahmed et al., 2022). On the contrary, some studies show the opposite pattern where fungal diversity is high in diseased soil, indicating that the variation of alpha-diversity may depend on the invading pathogen and the crop plant (Li et al., 2022). In this study, the PCoA revealed significant differences in the composition of fungal communities between asymptomatic and symptomatic Welsh onion plants of both phyllosphere and rhizosphere in each field. Similar differences in the beta-diversity were also observed for cotton, cucumber, rice, tobacco etc. in recent investigations (Huang et al., 2021; Wei et al., 2021; Jiang et al., 2023; Yang et al., 2023).

To the best of our knowledge, this is the first report on the core members of the Welsh onion mycobiome and the fungal communities in the disease microenvironment of leaf blight. These results will contribute to the identification of important fungal taxa influencing the development and spreading of leaf blight symptoms of Welsh onion in Taiwan. Further studies are needed to investigate the effect of abiotic conditions (growing season and agricultural practices) on fungal community variations in symptomatic plants, as these factors seem to play an important role in the development of leaf blight symptoms in Welsh onion.

## Data availability statement

The datasets presented in this study can be found in online repositories. The names of the repository/repositories and accession number(s) can be found below: https://www.ncbi.nlm.nih.gov/, PRJNA1022315.

## Author contributions

HJ: Data curation, Formal analysis, Investigation, Methodology, Visualization, Writing – original draft. H-XC: Writing – review and editing. SK: Formal analysis, Writing – review and editing. S-HY: Writing – review and editing. DH: Formal analysis, Writing – original draft. KA: Writing – review and editing. P-YL: Formal analysis, Writing – review and editing. MS: Writing – review and editing. HA: Conceptualization, Resources, Supervision, Writing – original draft, Writing – review and editing.
