# Supplementary material for: Metagenomic insight to apprehend the fungal communities associated with leaf blight of Welsh onion in Taiwan
Source: Front Plant Sci. 2024 Feb 29;15:1352997. doi: 10.3389/fpls.2024.1352997 (PMC10941342; doi:10.3389/fpls.2024.1352997)
Supplement: Supplementary file 1 [file DataSheet_1.pdf]

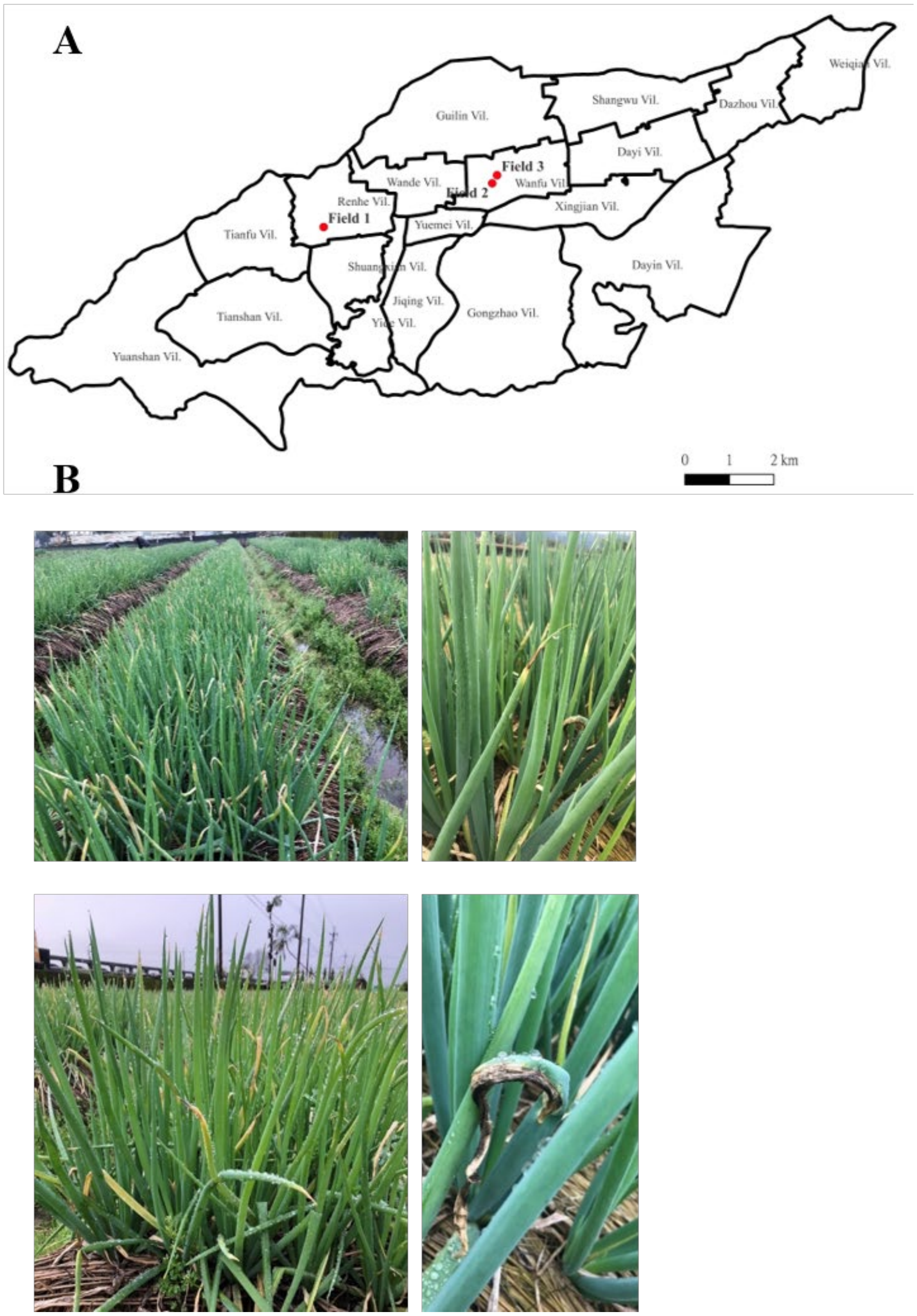

Figure S1 (A) Field locations in Sanxing. **(B)** Natural symptoms of leaf blight observed in Welsh onion fields.

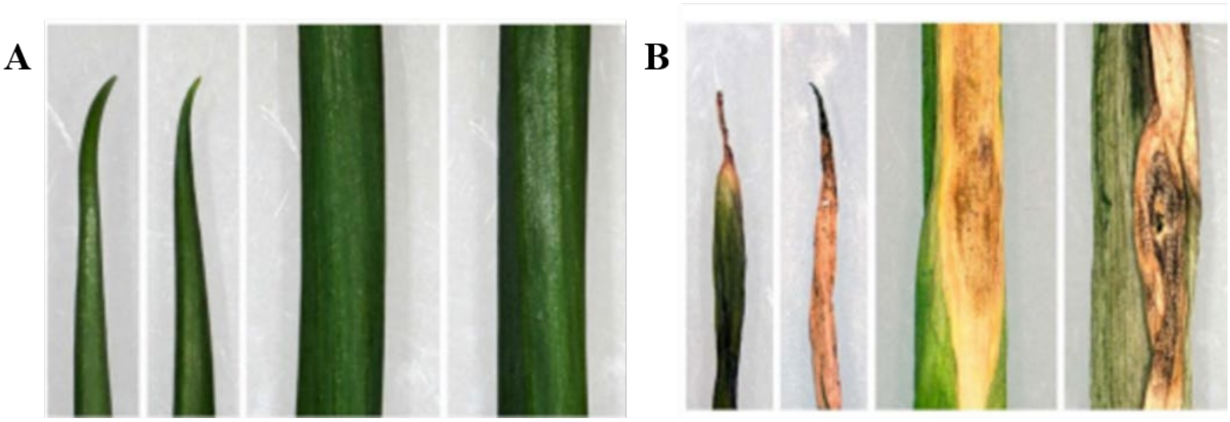

Figure S2 Welsh onion plants sampled during the study. (**A)** Healthy Welsh onion leaves without any symptoms of leaf blight. **(B)** Diseased Welsh onion leaves with leaf blight symptoms.

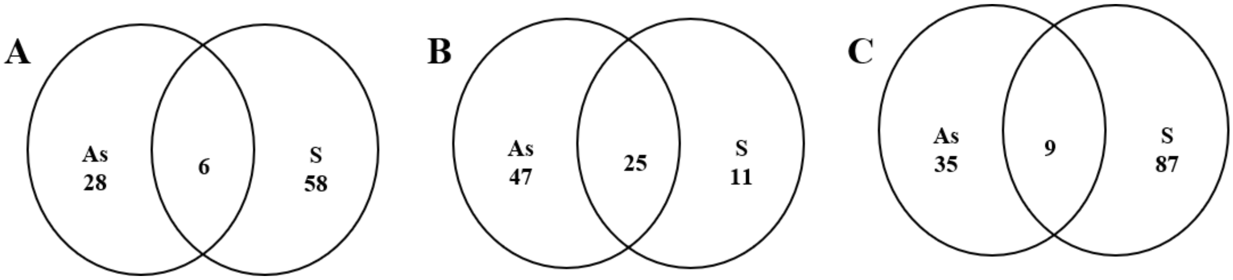

Figure S3 Venn-diagram illustrating the unique and shared ASVs between asymptomatic and symptomatic Welsh onion leaves in, **(A)** Field 1 **(B)** Field 2 and **(C)** Field 3; Asymptomatic leaves (AS) and Symptomatic leaves (S).

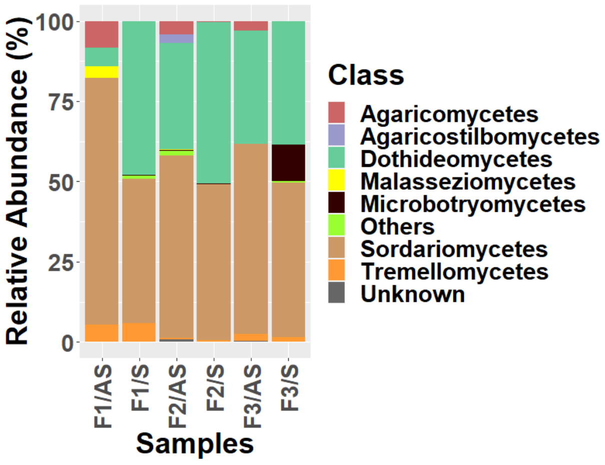

Figure S4 Relative abundance of the fungal communities in Welsh onion leaves. Stacked bar charts showing the relative abundance at class level divided according to the plant condition and the field. Taxa whose abundance was < 0.25% have been grouped into 'Others' category; taxa that did not classify at that specific taxonomic level was grouped into the category 'Unknown'; Field 1 (F1), Field 2 (F2), Field 3 (F3), Asymptomatic leaves (AS) and Symptomatic leaves (S).

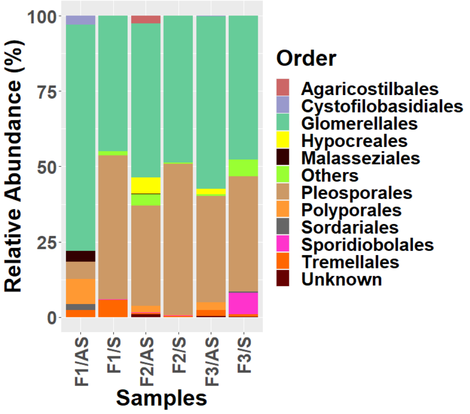

Figure S5 Relative abundance of the fungal communities in Welsh onion leaves. Stacked bar charts show the relative abundance at order level, divided according to the plant condition and the field. Taxa whose abundance was < 0.25% have been grouped into 'Others' category; taxa that did not classify at that specific taxonomic level was grouped into the category 'Unknown'; Field 1 (F1), Field 2 (F2), Field 3 (F3), Asymptomatic leaves (AS) and Symptomatic leaves (S).

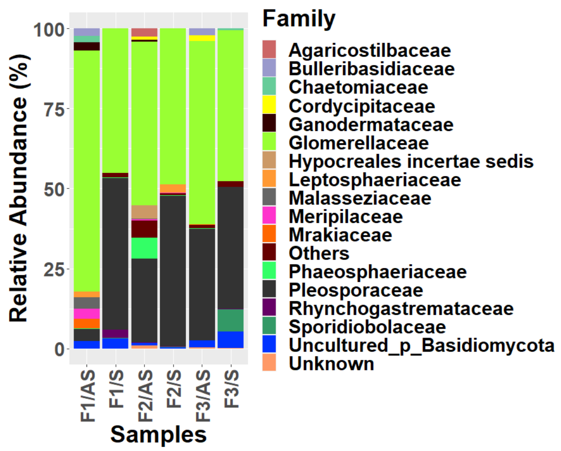

Figure S6 Relative abundance of the fungal communities in Welsh onion leaves. Stacked bar charts show the relative abundance at family level, divided according to the plant condition and the field. Taxa whose abundance was < 0.25% have been grouped into ‘Others’ category; taxa that did not classify at that specific taxonomic level was grouped into the category ‘Unknown’; Field 1 (F1), Field 2 (F2), Field 3 (F3), Asymptomatic leaves (AS) and Symptomatic leaves (S).

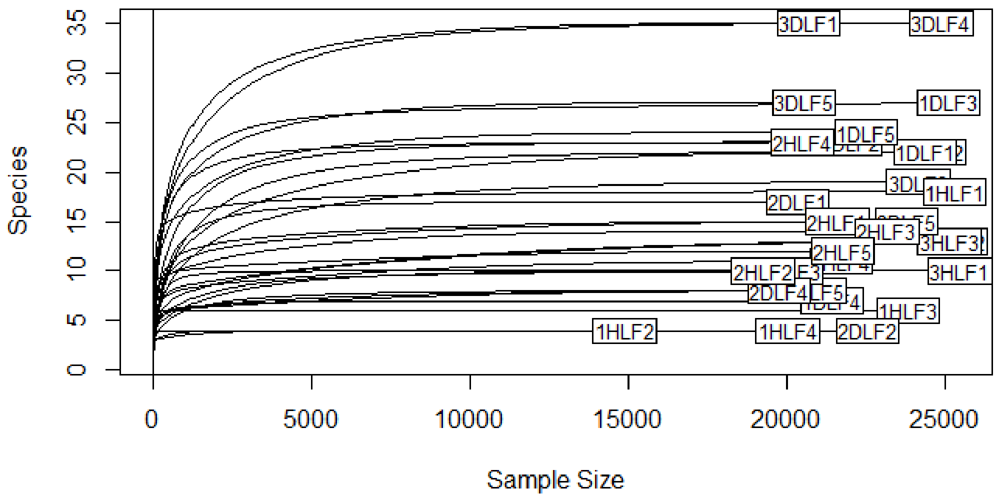

Figure S7 Rarefaction curves showing the relationship between the cumulative number of fungal ASVs in Welsh onion leaves and the sequencing intensity.

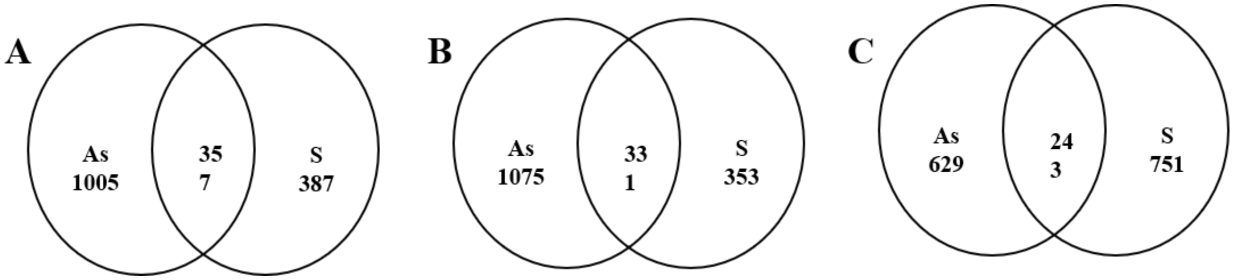

Figure S8 Venn-diagram illustrating the unique and shared ASVs between rhizosphere of asymptomatic and symptomatic Welsh onion plants in, **A**, Field 1, **B,** Field 2 and **C**, Field 3; Asymptomatic leaves (AS) and Symptomatic samples (S).

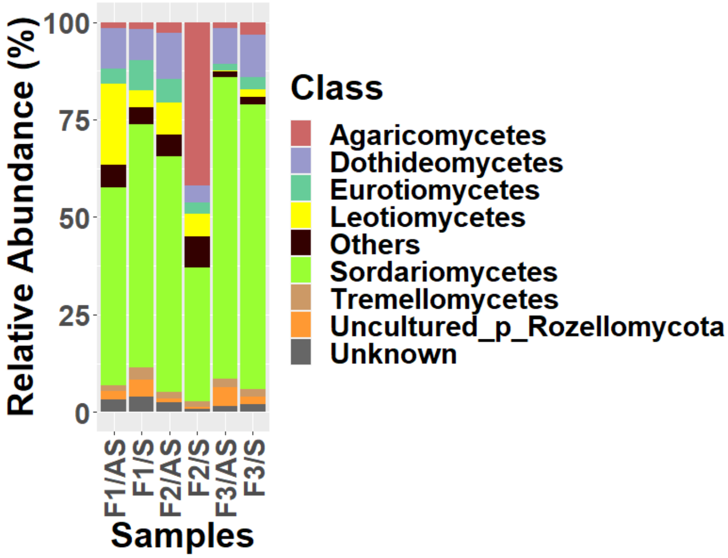

Figure S9 Relative abundance of the fungal communities in Welsh onion rhizosphere. Stacked bar charts show the relative abundance at class level divided according to the plant condition and the field. Taxa whose abundance was < 1% have been grouped into 'Others' category; taxa that did not classify at that specific taxonomic level was grouped into the category 'Unknown'; Field 1 (F1), Field 2 (F2), Field 3 (F3), Asymptomatic leaves (AS) and Symptomatic leaves (S).

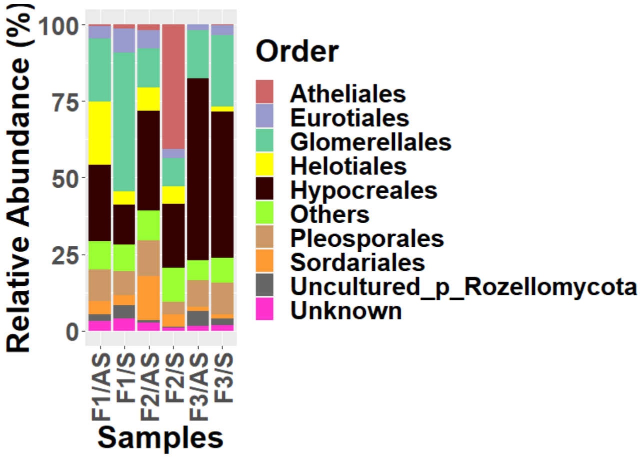

Figure S10 Relative abundance of the fungal communities in Welsh onion rhizosphere. Stacked bar charts show the relative abundance at order level divided according to the plant condition and the field. Taxa whose abundance was < 1% have been grouped into 'Others' category; taxa that did not classify at that specific taxonomic level was grouped into the category 'Unknown'; Field 1 (F1), Field 2 (F2), Field 3 (F3), Asymptomatic leaves (AS) and Symptomatic leaves (S).

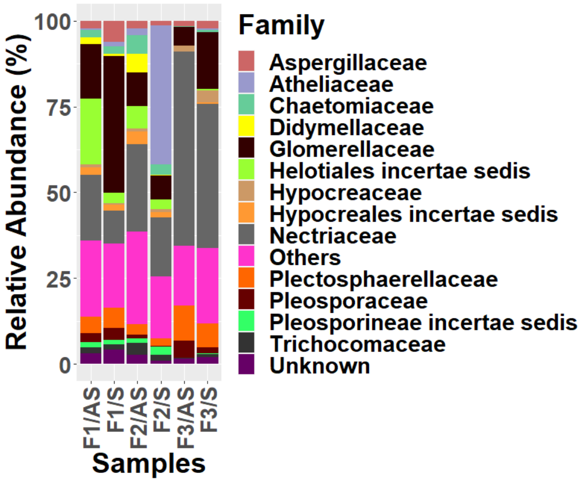

Figure S11 Relative abundance of the fungal communities in Welsh onion rhizosphere. Stacked bar charts show the relative abundance at family level divided according to the plant condition and the field. Taxa whose abundance was < 1% have been grouped into 'Others' category; taxa that did not classify at that specific taxonomic level was grouped into the category 'Unknown'; Field 1 (F1), Field 2 (F2), Field 3 (F3), Asymptomatic leaves (AS) and Symptomatic leaves (S).

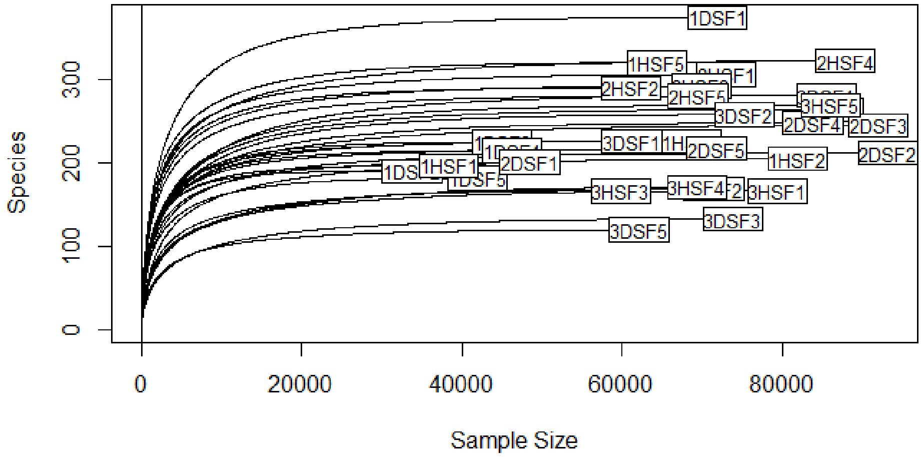

Figure S12 Rarefaction curves based on the number of fungal species reached a plateau when increasing the number of samples, indicating that the sampling depth was sufficient to cover actual fungal diversity within the samples.

Table S1 Plant part, plant condition, and the sample names used in the study.

Plant part	Plant condition	Location	Sample name
Leaves	Symptomatic	Field 1	1DLF
	Asymptomatic	Field 1	1HLF
	Symptomatic	Field 2	2DLF
	Asymptomatic	Field 2	2HLF
	Symptomatic	Field 3	3DLF
	Asymptomatic	Field 3	3HLF
Rhizosphere	Symptomatic	Field 1	1DSF
	Asymptomatic	Field 1	1HSF
	Symptomatic	Field 2	2DSF
	Asymptomatic	Field 2	2HSF
	Symptomatic	Field 3	3HSF
	Asymptomatic	Field 3	3HSF

Table S2 List of primers used in two step PCR of ITS1 region.

	Primer name	Primer sequences	Size
First step	NSA3-F	AAACTCTGTCGTGCTGGGGATA	1136 bp
	NLC2-R	GAGCTGCATTCCCAAACAACTC	
Second step	ITS1-F-KYO1	CTHGGTCATTTAGAGGAASTAA	200~300 bp
	ITS2	GCTGCGTTCTTCATCGATGC	

Table S3 PCR conditions of two step PCR of ITS1 region.

	First step			Second step		
Initiation	94℃	5 min		95℃	3 min	
Denaturation	94℃	30 sec	35 cycles	98℃	20 sec	25 cycles
Annealing	55℃	30 sec		57.5℃	20 sec	
Extension	72℃	30 sec		72℃	20 sec	
Final extension	72℃	5 min		72℃	3 min	

Table S4 Sequence details after denoising by DADA2 in Qiime2 (Phyllosphere).

Sample	No. of input sequences	No. of filtered sequences	Input sequences filtered (%)	No. of denoised sequences	No. of merged sequences	Input sequences merged (%)	No. of non-chimeric sequences	Input sequences non-chimeric (%)
1DL1	34642	27487	79.35	27452	27180	78.46	24548	70.86
1DL2	30142	24141	80.09	24082	23709	78.66	22175	73.57
1DL3	32419	26766	82.56	26737	26322	81.19	25095	77.41
1DL4	28504	22850	80.16	22776	22647	79.45	21403	75.09
1DL5	29997	24103	80.35	24039	23788	79.3	22502	75.01
1HL1	34771	27591	79.35	27477	27172	78.15	25607	73.64
1HL2	30499	23679	77.64	23642	23473	76.96	22890	75.05
1HL3	33064	25953	78.49	25885	25541	77.25	24887	75.27
1HL4	29949	22273	74.37	22239	21901	73.13	20343	67.93
1HL5	31533	22126	70.17	22080	21842	69.27	21052	66.76
2DL1	31326	22557	72.01	22536	22131	70.65	20379	65.05
2DL2	29925	23781	79.47	23751	23494	78.51	22519	75.25
2DL3	27523	20937	76.07	20900	20787	75.53	20168	73.28
2DL4	25973	20857	80.3	20844	20663	79.56	19789	76.19
2DL5	31818	24539	77.12	24527	24344	76.51	23774	74.72
2HL1	34133	26025	76.25	25964	25729	75.38	25174	73.75
2HL2	32541	25205	77.46	25163	25020	76.89	24686	75.86
2HL3	31271	24588	78.63	24522	24289	77.67	23431	74.93
2HL4	27901	21389	76.66	21284	21152	75.81	20705	74.21
2HL5	32415	24471	75.49	24408	24192	74.63	23168	71.47
3DL1	29077	22768	78.3	22726	22596	77.71	20690	71.16
3DL2	30449	25564	83.96	25481	25330	83.19	24769	81.35
3DL3	31694	25420	80.2	25369	25127	79.28	24186	76.31
3DL4	34266	26736	78.02	26672	26468	77.24	25163	73.43
3DL5	26893	22367	83.17	22310	21999	81.8	20550	76.41
3HL1	33546	27050	80.64	27033	26840	80.01	26229	78.19
3HL2	34842	26684	76.59	26592	26351	75.63	25506	73.2
3HL3	32771	26183	79.9	26159	25713	78.46	25191	76.87

3HL4	28503	22895	80.32	22889	22566	79.17	21879	76.76
3HL5	25839	20719	80.18	20706	20448	79.14	20187	78.13

Table S5 Global network property comparison of the fungal communities between asymptomatic and symptomatic Welsh onion leaves.

**Global network properties [a]**	**asymptomatic**	**symptomatic**	**abs.diff.**	**p-value**
Number of components	23.000	12.000	11.000	0.170829
Clustering coefficient	0.734	0.464	0.270	0.068931
Modularity	0.754	0.408	0.346	0.0.004995**
Positive edge percentage	89.286	73.770	15.515	0.147852
Edge density	0.031	0.068	0.037	0.046953 *
Natural connectivity	0.031	0.034	0.003	0.364635
Vertex connectivity	1.000	1.000	0.000	1.000000
Edge connectivity	1.000	1.000	0.000	1.000000
Average dissimilarity [b]	0.861	0.951	0.090	0.037962*
Average path length [c]	1.595	1.657	0.063	0.897103

[a] Group differences are compared with the Sparse Correlations for Compositional (SparCC) method via NetCoMi netCompare. Permutation tests were done with 1,000 permutations and adjusted with 'adaptBH' at cores of 4, seed of 123456. The P value for testing the null hypothesis HO: |diff| = 0.

[b] Dissimilarity = 1 - edge weight

[c] Units with average dissimilarity.

Significance codes: ***: 0.001, **: 0.01, *: 0.05, .: 0.1

Table S6 Jaccard index values of the phyllosphere fungal networks.

**Properties**	**j**	**P (J ≤ j)**	**P (J ≥ j)**
Degree	0.105	0.024021*	0.995264
Betweenness centrality	0.143	0.105334	0.972596
Closeness centrality	0.158	0.078659	0.975979
Eigenvector centrality	0.222	0.231072	0.898335
Hub taxa	0.000	0.087791	1.000000

[a] Index values j express the similarity of the sets of most central nodes and of the sets of hub taxa between the two networks. "Most central" nodes are those with a centrality value above the empirical 75% quantile. Jaccard's index is 0 if the sets are completely different and 1 for exactly equal sets. P(J≤j) is the probability that Jaccard's index takes a value less than or equal to the calculated index j for the present total number of taxa in both sets; P(J≥ j) is defined analogously.

Table S7 Sequence details after denoising by DADA2 in Qiime2 (Rhizosphere).

Sampl e	No. of input sequenc es	No. of filtered sequenc es	Input sequenc es filtered (%)	No. of denoise d sequenc es	No. of merged sequenc es	Input sequenc es merged (%)	No. of non-chimeric sequenc es	Input sequenc es non-chimeric (%)
1DSF 1	86345	75991	88.01	74922	71848	83.21	68028	78.79
1DSF 2	117813	102986	87.41	101678	98010	83.19	93681	79.52
1DSF 3	99564	86557	86.94	85634	83233	83.6	80648	81
1DSF 4	102335	89237	87.2	88193	84774	82.84	82312	80.43
1DSF 5	95553	82659	86.51	81695	78746	82.41	75993	79.53
1HSF 1	101149	89350	88.34	87699	83431	82.48	80417	79.5
1HSF 2	101428	92092	90.8	91203	88399	87.15	85283	84.08

1HSF 3	118995	106713	89.68	105468	102154	85.85	97970	82.33
1HSF 4	102150	92036	90.1	90819	88836	86.97	84526	82.75
1HSF 5	89328	79110	88.56	78016	74133	82.99	71467	80.01
2DSF 1	63444	57110	90.02	56587	54416	85.77	53069	83.65
2DSF 2	117571	106272	90.39	105467	102699	87.35	100221	85.24
2DSF 3	115491	104266	90.28	103547	100716	87.21	98547	85.33
2DSF 4	106547	96679	90.74	95976	93490	87.75	91137	85.54
2DSF 5	96731	86076	88.98	85414	80226	82.94	78230	80.87
2HSF 1	95681	85608	89.47	84285	81002	84.66	78169	81.7
2HSF 2	84653	75294	88.94	73843	70847	83.69	68558	80.99
2HSF 3	89708	80862	90.14	79860	77268	86.13	75359	84
2HSF 4	115369	103234	89.48	101947	98024	84.97	95130	82.46
2HSF 5	90751	81351	89.64	80290	77566	85.47	75013	82.66
3DSF 1	83404	75211	90.18	74403	72100	86.45	69679	83.54
3DSF 2	94972	90095	94.86	88570	85148	89.66	82096	86.44
3DSF 3	98431	91052	92.5	90461	88772	90.19	81202	82.5
3DSF 4	110607	99000	89.51	98069	95759	86.58	92117	83.28
3DSF 5	84338	77930	92.4	77098	75562	89.59	70447	83.53
3HSF 1	106331	97114	91.33	95837	92695	87.18	85145	80.08
3HSF 2	90374	83451	92.34	82373	79531	88	76032	84.13
3HSF 3	78813	71844	91.16	71063	69574	88.28	65969	83.7
3HSF 4	89497	82396	92.07	81443	78301	87.49	73495	82.12
3HSF 5	100284	93242	92.98	92503	90626	90.37	88413	88.16

Table S8 Global network property comparison of the fungal communities between asymptomatic and symptomatic Welsh onion rhizosphere.

**Global network properties** [a]	**asymptomatic**	**symptomatic**	**abs.diff.**	**p-value**
Number of components	2.000	1.000	1.000	0.613387
Clustering coefficient	0.742	0.664	0.078	0.404595
Modularity	0.006	0.055	0.048	0.164835
Positive edge percentage	50.036	49.070	0.966	0.457542
Edge density	0.441	0.357	0.084	0.287712
Natural connectivity	0.170	0.124	0.046	0.194805
Vertex connectivity	3.000	1.000	2.000	0.185814
Edge connectivity	3.000	1.000	2.000	0.184815
Average dissimilarity [b]	0.845	0.880	0.035	0.210789
Average path length [c]	1.081	1.150	0.070	0.326673

[a] Group differences are compared with the Sparse Correlations for Compositional (SparCC) method via NetCoMi netCompare. Permutation tests were done with 1,000 permutations and adjusted with 'adaptBH' at cores of 4, seed of 123456. The P value for testing the null hypothesis HO: |diff| = 0.

[b] Dissimilarity = 1 - edge weight

[c] Units with average dissimilarity.

Significance codes: ***: 0.001, **: 0.01, *: 0.05, .: 0.1

Table S9 Jaccard index values of the rhizosphere fungal networks.

**Properties**	**j**	**P (J ≤ j)**	**P (J ≥ j)**
Degree	0.370	0.734226	0.410781
Betweenness centrality	0.143	0. 009891**	0.997076
Closeness centrality	0.250	0. 210559	0.884673
Eigenvector centrality	0.111	0. 002175**	0.999511
Hub taxa	0.000	0.039018**	1.000000

[a] Index values j express the similarity of the sets of most central nodes and of the sets of hub taxa between the two networks. "Most central" nodes are those with a centrality value above the empirical 75% quantile. Jaccard's index is 0 if the sets are completely different and 1 for exactly equal sets. P(J≤j) is the probability that Jaccard's index takes a value less than or equal to the calculated index j for the present total number of taxa in both sets; P(J≥ j) is defined analogously.
